# A20 restricted PDCoV release through negative regulation of PANoptosis

**DOI:** 10.1128/mbio.00081-26

**Published:** 2026-03-03

**Authors:** Chunyu Lu, Xiaofeng Xue, Zhuoqi Chen, Wei Wang, Rongli Guo, Min Sun, Baochao Fan, Bin Li, Jizong Li

**Affiliations:** 1 College of Veterinary Medicine, Hebei Agricultural University 74562, Baoding, China; 2 Institute of Veterinary Medicine, Jiangsu Academy of Agricultural Sciences, Key Laboratory of Veterinary Biological Engineering and Technology Ministry of Agriculture; Jiangsu Key Laboratory for Food Quality and Safety-State Key Laboratory Cultivation Base of Ministry of Science and Technology 117941, Nanjing, China; 3 College of Veterinary Medicine, Nanjing Agricultural University 70578, Nanjing, China; 4 Jiangsu Co-Innovation Center for the Prevention and Control of Important Animal Infectious Disease and Zoonose, Yangzhou University 38043, Yangzhou, China; Ulm University Medical Center, Ulm, Baden-Württemberg, Germany

**Keywords:** coronavirus, PDCoV, PANoptosis, A20, viral release, zoonosis

## Abstract

Coronavirus infections can trigger multiple modes of cell death, leading to severe infectious diseases. The process is modulated by host factors with mechanisms yet to be fully elucidated. Here, we first demonstrated that the host factor A20 regulated PANoptosis during porcine deltacoronavirus (PDCoV) infection, thereby contributing to the antiviral defense response. We found that PDCoV could induce PANoptosis in intestinal epithelial cells, which facilitates the extracellular release of viral particles through this form of programmed cell death. A20 restricted the PANoptosome assembly and downstream death signaling by targeting RIPK3 ubiquitin chains for degradation. Consequently, loss of A20 exacerbated cell lysis and enhanced the release of viral particles, although this effect does not alter viral entry or replication. We further established that PDCoV-induced PANoptosis-dependent release was driven by osmotic imbalance resulting from membrane pore formation mediated by GSDMD and MLKL, rather than by direct transmembrane egress of viral particles. Transwell models showed that pharmacological inhibition of the pore-forming activities of GSDMD and MLKL reduced viral dissemination and preserved epithelial barrier integrity. These findings advance our understanding of enteric coronavirus pathogenesis and suggest that the A20-PANoptosis axis represents a potential target for antiviral intervention.

**IMPORTANCE:**

Coronaviruses have repeatedly posed significant threats to both human and animal health. Here, we used porcine deltacoronavirus (PDCoV), a highly enterotropic zoonotic pathogen, to uncover a novel mechanism by which coronaviruses exploit PANoptosis to facilitate viral egress. We demonstrate that PDCoV infection triggers PANoptosis in intestinal epithelial cells, leading to plasma membrane rupture and subsequent viral release. Importantly, we identified the host ubiquitin-editing enzyme A20 as a critical negative regulator of this process. A20 restricts PANoptosome assembly by specifically deubiquitinating RIPK3, thereby limiting cell lysis and suppressing viral dissemination without affecting viral replication. Our findings offer fundamental insights into coronavirus-host interactions and highlight the therapeutic potential of targeting lytic cell death to combat viral dissemination.

## INTRODUCTION

Coronaviruses (CoVs) are enveloped, positive-sense single-stranded RNA viruses, classified into four genera: α, β, γ, and δ coronavirus. They can infect a wide range of animals and humans, causing respiratory, gastrointestinal, and neurological diseases (1, 2). Porcine deltacoronavirus (PDCoV), a member of the genus Deltacoronavirus, was first identified in Hong Kong, China, in 2012 (3). Since then, it has spread globally and poses a significant threat to the swine industry (4–6). PDCoV primarily affects suckling piglets, leading to acute gastroenteritis. Clinical signs include severe diarrhea, dehydration, and high mortality (7). PDCoV exhibits strong enterotropism, targeting intestinal epithelial cells and causing villous atrophy and disruption of the intestinal barrier (8). Although viral nucleic acids are detectable in tissues such as the lungs and liver, no significant pathological damage has been observed in these organs (8). Recent studies have shown that PDCoV can undergo cross-species transmission, infecting animals such as mice, cattle, and chickens (9–12). More notably, in 2021, Burbelo et al. detected PDCoV-specific nucleic acid sequences in the plasma of three febrile children in Haiti (13). This discovery confirms the virus’s ability to cross species barriers and raises serious public health concerns about its potential zoonotic risk.

PANoptosis is an inflammatory form of programmed cell death (PCD) that integrates key features of apoptosis, pyroptosis, and necroptosis. Its central mechanism involves the dynamic assembly of the PANoptosome complex. This molecular platform coordinates crosstalk between multiple PCD pathways by recruiting key proteins, including caspase-8, RIPK3, and ASC (14). The activation and composition of the PANoptosome depend on the stimulus, timing, and cell type. To date, ZBP1, AIM2, RIPK1, and NLRP12 have been identified as drivers of PANoptosome assembly (15). Among these, ZBP1 mediates antiviral responses by recognizing viral RNA and initiating PANoptosis. For instance, PANoptosis activation contributes to host defense against lethal infections caused by IAV and HSV-1 (16, 17). However, PANoptosis is not always advantageous for host survival. Excessive activation of PANoptosis is a major contributor to cytokine storm and multi-organ failure during SARS-CoV-2 infection (18–20). Similar pathogenic outcomes have been observed in infections with MERS-CoV and MHV (21, 22). Although the role of PANoptosis in respiratory coronavirus infections has been reported, its regulatory mechanism in enteric coronavirus remains to be elucidated. PDCoV, as a highly enteric coronavirus with cross-species transmission, is an ideal model for studying the PANoptosis induced by enteric coronaviruses. Whether it forms a functional PANoptosome and triggers the complete PANoptotic program remains to be determined. Elucidating the role of PANoptosis in its pathogenesis may reveal molecular targets for antiviral therapy and provide critical insights into its zoonotic risk.

A20 (TNFAIP3) is a multifunctional ubiquitin-editing enzyme that regulates inflammatory and cell death signaling pathways through the coordinated activity of its distinct structural domains (23). The N-terminus of A20 contains an ovarian tumor (OTU) deubiquitinase domain that removes K63-linked polyubiquitin chains (24, 25). The C-terminus comprises seven ZnF domains (ZnF1-7). ZnF4 has E3 ubiquitin ligase activity, while ZnF7 specifically binds to linear ubiquitin chains (M1-linked) (26, 27). This dual enzymatic activity allows A20 to precisely regulate the ubiquitination of inflammation-associated signaling molecules. As a critical negative regulator of the NF-κB pathway, A20 suppresses inflammatory signaling by modulating the ubiquitination of key mediators, including RIPK1 and TRAF6 (28, 29). In addition, A20 plays a vital role in regulating PCD. It modulates necroptosis and pyroptosis by regulating the ubiquitination of critical complexes such as the Ripoptosome, complex II, and the NLRP3 inflammasome (30, 31). This regulatory capacity, together with its inhibitory effects on Toll-like receptor and TNF receptor signaling (32, 33), highlights A20 as a central node in maintaining immune homeostasis and promoting cell survival.

This study uncovered the molecular mechanism by which PDCoV promoted viral release through the induction of PANoptosis in intestinal epithelial cells. We further showed that the host factor A20 counteracted this process by selectively deubiquitinating RIPK3, thereby suppressing PANoptosome formation and inhibiting PANoptosis. Through this regulation, A20 preserved intestinal mucosal barrier integrity and significantly limited intercellular viral transmission. These findings provided new insights into the crosstalk between PDCoV and host cell death pathways and supported PANoptosis as a potential target for antiviral intervention.

## RESULTS

### PDCoV triggered PANoptosis in porcine jejunal epithelial cell

**Fig 1:**
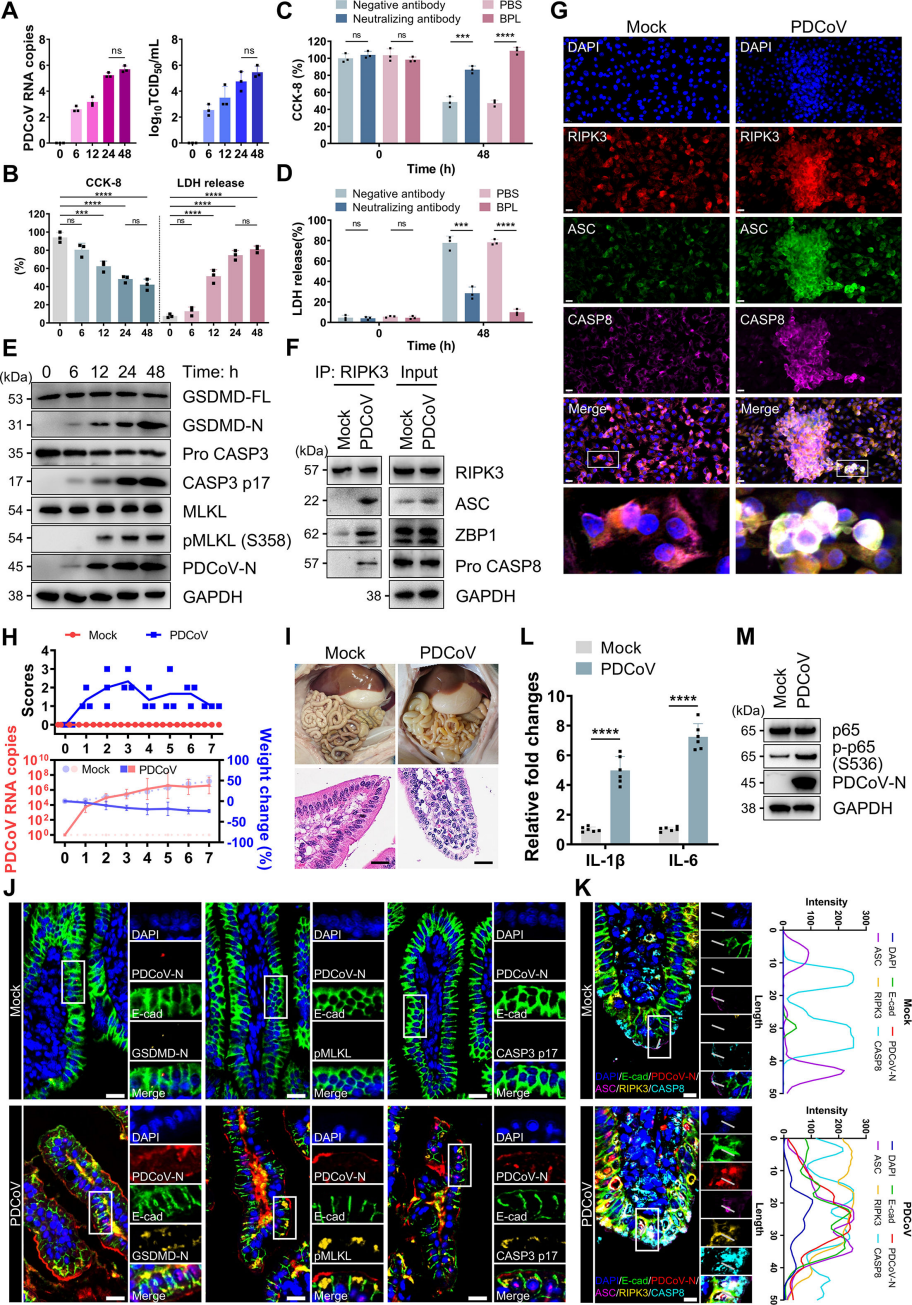
PDCoV triggered PANoptosis in intestinal epithelial cells. (**A**) qRT-PCR quantification of PDCoV copy numbers in cytoplasmic lysates of IPEC-J2 (MOI of 0.5). TCID50 assay of PDCoV titers. *n* = 3. (**B**) Cell viability and cytotoxicity of IPEC-J2 cells at the indicated time points post-infection with PDCoV (MOI of 0.5). *n* = 3. (**C and D**) Cell viability (**C**) and cytotoxicity (**D**) of IPEC-J2 at indicated times post-infection with PDCoV (MOI of 0.5) after antibody-mediated neutralization or inactivation. *n* = 3. (**E**) Immunoblot analysis of the cleavage or phosphorylation of GSDMD, caspase-3, and MLKL, along with the expression of PDCoV N protein. Blots for GAPDH served as loading controls. (**F**) Co-IP of RIPK3 with ASC, ZBP1, and pro caspase-8 at 24 hpi. (**G**) Representative immunofluorescence images showing co-localization of RIPK3, ASC, and pro-caspase-8 in IPEC-J2 at 24 hpi with PDCoV (MOI of 0.5). Scale bar, 20 μm. (**H**) Fecal consistency scores were recorded (above). Viral shedding (red) and body weight (blue) were recorded (below). *n* = 3. (**I**) Pathological examination and H&E-stained jejunum sections. Scale bars, 20 μm. (**J**) Representative immunofluorescence images of jejunal tissue sections from piglets at 48 hpi. Nuclei were shown in blue, pMLKL or GSDMD-N or caspase-3 p17 in ochre, the epithelial cell marker E-cadherin in green, and PDCoV in red. Scale bar, 20 μm. (**K**) Representative immunofluorescence images showing colocalization of PANoptosome components in jejunal tissues. Nuclei were shown in blue, E-cadherin in green, PDCoV in red, ASC in purple, RIPK3 in ochre, and pro-caspase-8 in light blue. Scale bar, 10 μm. Line plots showed fluorescence intensity profiles of 50 pixels for co-localization analysis in each image. (**L**) mRNA expression levels of IL-1β and IL-6 in jejunal tissues of piglets at 48 hpi. *n* = 6. (**M**) Immunoblot analysis of PDCoV-N and total and phosphorylated p65. Blots for GAPDH served as loading controls. All experiments were representative of three or more independent experiments. Bars indicated mean + SD. **** *P* < 0.0001, *** *P* < 0.0005, ** *P* < 0.005, and * *P* < 0.05; ns, no significant difference.

To evaluate the effect of PDCoV infection on porcine intestinal epithelial cell survival, IPEC-J2 was used as an *in vitro* model. Following PDCoV infection of IPEC-J2, the viral load and titer increased significantly in a time-dependent manner (Fig. 1A). Cell viability assays showed a time-dependent decrease in IPEC-J2 cells’ viability following PDCoV infection (Fig. 1B), along with a significant increase in cytotoxicity (Fig. 1B). To confirm that the observed cytotoxicity was attributable to viral infection rather than confounding factors, PDCoV was either neutralized with specific antibodies or inactivated with propiolactone (BPL), with negative antibodies and PBS serving as controls. The results indicated that antibody-mediated neutralization markedly increased IPEC-J2 cell viability (Fig. 1C) and concomitantly reduced cytotoxicity (Fig. 1D). In contrast, inactivated virus did not cause significant impairment of cell survival (Fig. 1C and D). These results suggested that IPEC-J2 was highly susceptible to PDCoV.

To further evaluate the potential induction of cell death by PDCoV infection in IPEC-J2, the cleavage of pyroptosis effector GSDMD (GSDMD-N), apoptosis marker caspase-3 (CASP3 p17), and the phosphorylation of necroptosis effector MLKL (pMLKL) were examined. Western blot (WB) analysis showed that PDCoV infection simultaneously activated all three forms of PCD (Fig. 1E). In fact, mild activation of GSDMD-N and CASP3 p17 was observed as early as 6 h post-infection (hpi; Fig. 1E), indicating early onset of pyroptosis and apoptosis. In contrast, significant phosphorylation of MLKL was observed at 12 hpi (Fig. 1E). This temporal pattern aligned with decreased cell viability and increased cytotoxicity (Fig. 1B), suggesting that the mode of cell death directly affected IPEC-J2 survival. Notably, no significant differences in viability or cytotoxicity were found between 24 and 48 hpi (Fig. 1B). Viral replication analysis based on PDCoV N protein showed that intracellular viral load peaked at 24 h and did not increase further by 48 h (Fig. 1A and E). Therefore, 24 hpi was chosen as the optimal time point for subsequent experiments.

PANoptosis involvement was next assessed by evaluating the formation of the PANoptosome complex. Co-immunoprecipitation (Co-IP) assays revealed that RIPK3 specifically interacted with caspase-8, ASC, and ZBP1 at 24 hpi (Fig. 1F). Subsequent colocalization analysis verified the formation of intracellular specks comprising these molecules (Fig. 1G). These findings confirm PANoptosome assembly in IPEC-J2, indicating activation of PANoptosis. To validate the *in vivo* relevance of this finding, a piglet infection model was established, and body weight and viral shedding were monitored over 7 days. Infected piglets exhibited a sustained decrease in body weight throughout the observation period, concurrent with pronounced diarrheal symptoms (Fig. 1H). Viral shedding analysis revealed detectable virus in feces as early as 24 hpi, with levels plateauing by 4 days post-infection (Fig. 1H). Histopathological examination at 48 hpi showed marked dilation of the small intestine, with thinning and translucency of the intestinal wall (Fig. 1I). The jejunal mucosal villi exhibited diffuse and severe fusion, accompanied by mild erythrocyte extravasation in the lamina propria (Fig. 1I), confirming the successful establishment of the piglet infection model. The activation of PANoptosis in intestinal epithelial cells was then examined. Compared with controls, intestinal epithelial cells from infected piglets showed strong expression of active caspase-3 (p17), GSDMD (GSDMD-N), and phosphorylated MLKL (pMLKL; Fig. 1J). Consistently, strong colocalization of RIPK3, caspase-8, and ASC was also observed (Fig. 1K). These results demonstrated that PDCoV induced PANoptosis in intestinal epithelial cells and that this process was conserved in both *in vitro* and *in vivo* settings.

Given the inflammatory nature of PANoptosis, we examined the inflammatory response in the jejunal tissue of piglets after PDCoV infection. The mRNA levels of IL-1β and IL-6 were significantly upregulated (Fig. 1L). Mechanistically, PDCoV infection increased phosphorylation of p65 at Ser356 (p-p65 S356) in the intestine (Fig. 1M), indicating activation of the NF-κB signaling pathway and transcription of pro-inflammatory cytokines.

### A20 involvement in the regulation of PANoptosis

**Fig 2:**
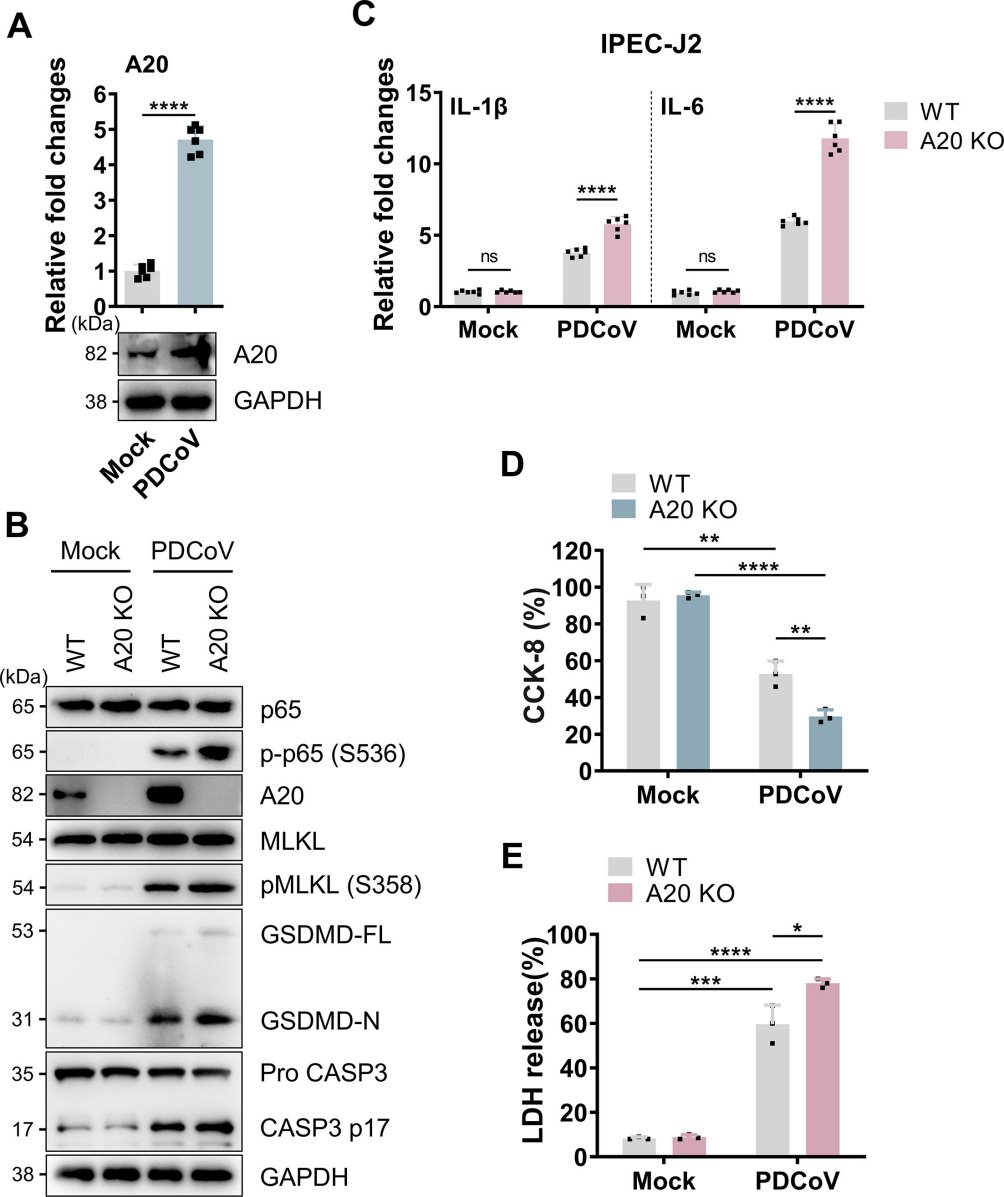
A20 regulated PDCoV-induced PANoptosis. (**A**) mRNA expression levels of A20 in IPEC-J2 cells at 24 hpi (MOI of 0.5). *n* = 6. Immunoblot analysis of A20. Blots for GAPDH served as loading controls. (**B**) Immunoblot analysis of total and phosphorylated p65 and MLKL, and cleaved GSDMD and caspase-3 in WT and A20 KO cells. Blots for GAPDH served as loading controls. (**C**) mRNA expression levels of IL-1β and IL-6 in WT and A20 KO cells at 24 hpi with PDCoV. *n* = 6. (**D**) Cell viability assay at 24 hpi in WT and A20 KO cells. *n* = 3. (**E**) Cytotoxicity assay at 24 hpi in WT and A20 KO cells. *n* = 3. All experiments were representative of three or more independent experiments. Bars indicated mean + SD. **** *P* < 0.0001, *** *P* < 0.0005, ** *P* < 0.005, and * *P* < 0.05, ns, no significant difference.

In our previous study, RNA-seq analysis showed that the NF-κB negative regulator A20 was significantly upregulated during PDCoV infection (34). This result was confirmed at both the mRNA and protein levels (Fig. 2A). A20 knockout (KO) cells were generated with CRISPR/Cas9 gene editing (Fig. 2B). As expected, A20 loss increased p65 phosphorylation (Fig. 2B) and cytokine transcription (Fig. 2C) after infection, confirming its canonical role. Surprisingly, A20 deletion made IPEC-J2 cells more sensitive to PDCoV, as shown by reduced viability (Fig. 2D) and elevated cytotoxicity (Fig. 2E). In A20 KO cells, phosphorylation of the necroptosis effector MLKL, cleavage of the pyroptosis effector GSDMD, and apoptosis effector Caspase-3 were also more pronounced than in wild-type (WT) cells (Fig. 2B). These enhanced PANoptotic features explain the greater sensitivity of A20 KO cells to PDCoV. These findings indicated that A20 restricted PDCoV-induced PANoptosis and protected host cells beyond its NF-κB regulatory function, thereby supporting cell survival.

### A20 regulated PANoptosome assembly by restricting RIPK3 ubiquitination

**Fig 3:**
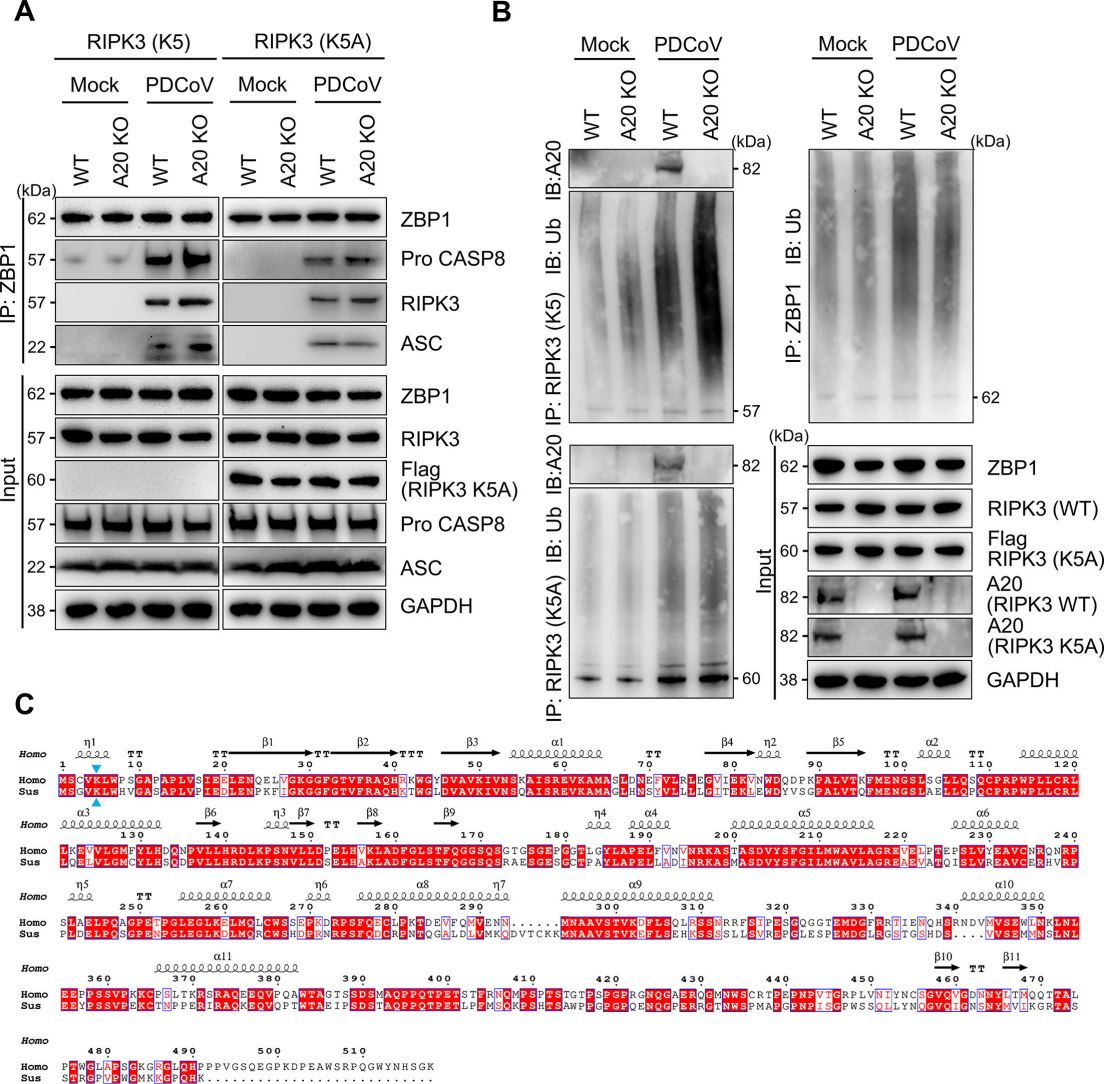
A20 targeted RIPK3 ubiquitination to regulate PANoptosome. (**A**) Co-immunoprecipitation analysis of interactions between ZBP1 and pro caspase-8, RIPK3, and ASC at 24 hpi in WT and A20 KO cells, under either wild-type (RIPK3 K5) or K5-mutant RIPK3 (RIPK3 K5A) backgrounds. (**B**) Immunoblot analysis of total and ubiquitinated levels of ZBP1, RIPK3 (K5), and RIPK3 (K5A) in WT and A20 KO cells. Co-IP analysis of RIPK3 (K5) or RIPK3 (K5A) with A20 in WT and A20 KO cells. Blots for GAPDH served as loading controls. (**C**) Sequence alignment of human and Sus scrofa RIPK3 proteins. Identical residues were shaded in red, and the conserved lysine residue K5 was indicated by a blue triangle. All experiments were representative of three or more independent experiments.

To elucidate the regulatory mechanism of A20 in PANoptosis, we examined the core PANoptosome components ZBP1 and RIPK3. Co-IP assays confirmed that ZBP1 formed a complex with caspase-8, RIPK3, and ASC, supporting its role in PANoptosome assembly (Fig. 3A). Compared with WT, A20 deficiency significantly promoted PANoptosome formation (Fig. 3A), suggesting that A20 negatively regulated PANoptosis by suppressing PANoptosome assembly. Further analysis showed a slight increase in ZBP1 ubiquitination after PDCoV infection, but no significant difference between WT and A20 KO (Fig. 3B). In contrast, RIPK3 ubiquitination was substantially enhanced in A20 KO following infection (Fig. 3B). K5 had been identified as a key ubiquitination site in human RIPK3 (35), and this residue was conserved in both porcine and human RIPK3 (Fig. 3C). To confirm the role of RIPK3 ubiquitination in A20-mediated PANoptosome assembly, we generated a lysine-to-alanine RIPK3 mutant (RIPK3 K5A) in IPEC-J2 cells (Fig. 3B). RIPK3 K5A significantly reduced PDCoV-induced RIPK3 ubiquitination (Fig. 3B). Notably, RIPK3 K5A abolished the enhanced ubiquitination seen in the absence of A20 (Fig. 3B), accompanied by a pronounced impairment of PANoptosome assembly following infection (Fig. 3A). The mutation also abolished the promoting effect of A20 deficiency on PANoptosome formation (Fig. 3A), indicating that A20 regulates assembly through RIPK3 ubiquitination. Pull-down assays revealed an interaction between A20 and RIPK3, which was weakened in the ubiquitination-defective RIPK3 K5A (Fig. 3B), suggesting that RIPK3 was a direct target of A20-mediated ubiquitination regulation. Together, these findings demonstrated that A20 restricted PANoptosome assembly by specifically regulating RIPK3 ubiquitination, thereby serving a critical cytoprotective role during PDCoV infection.

### A20 restricted PDCoV release by regulating PANoptosis

**Fig 4:**
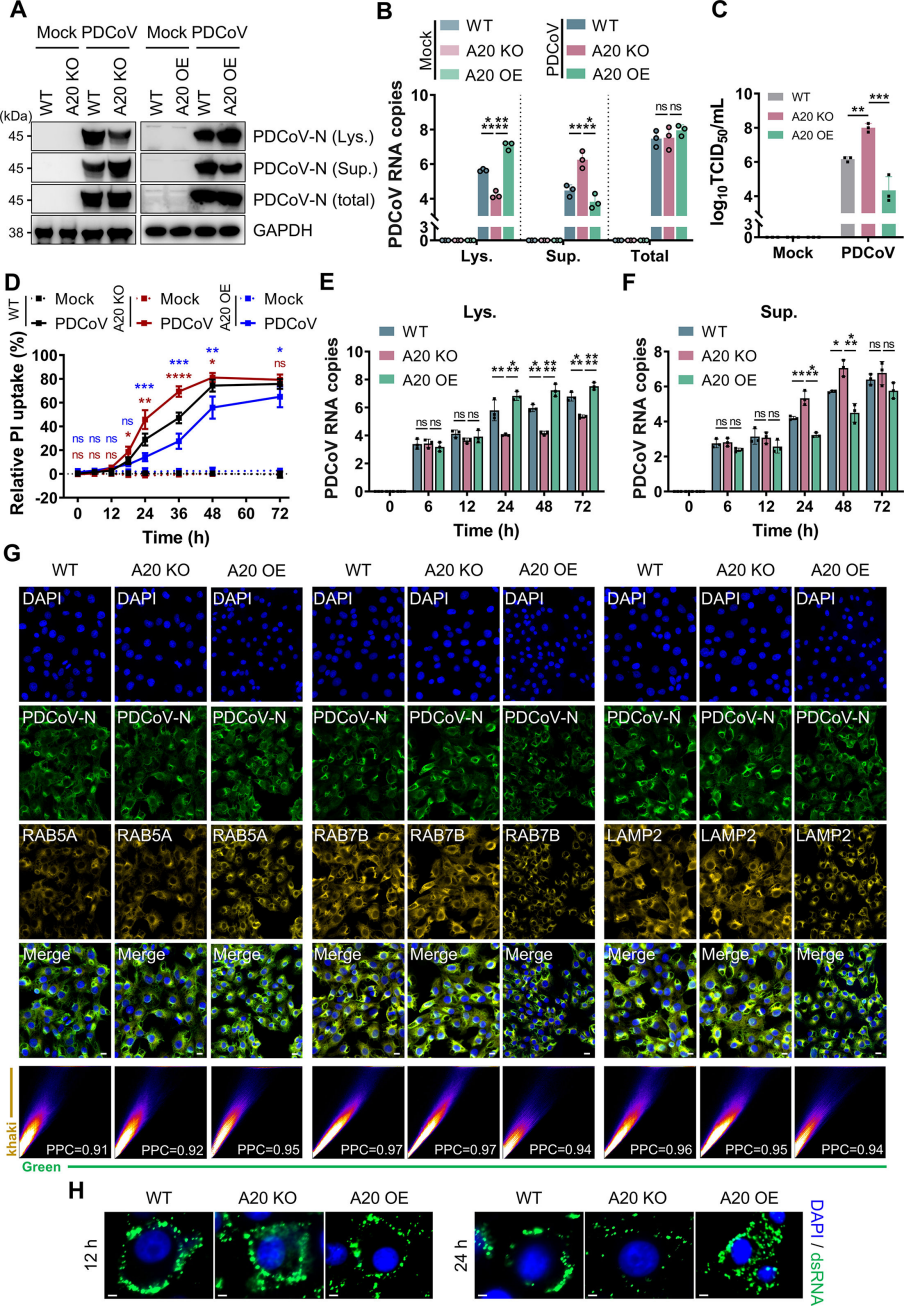
A20 deficiency promoted PDCoV release. (**A**) Immunoblot analysis of PDCoV N protein levels in cytoplasmic lysates (Lys.) and supernatants (Sup.) of WT, A20 KO, and A20 OE cells at 24 hpi. Blots for GAPDH served as loading controls. (**B**) qRT-PCR quantification of PDCoV N gene copy numbers in cytoplasmic lysates (Lys.) and supernatants (Sup.) of WT, A20 KO, and A20 OE cells at 24 hpi (MOI of 0.5). *n* = 3. (**C**) TCID_50_ assay of viral titers released from WT, A20 KO, and A20 OE cells at 24 hpi (MOI of 0.5). *n* = 3. (**D**) Kinetics of PI-permeable cell death in WT, A20 KO, and A20 OE cells following PDCoV infection (MOI of 0.5). *n* = 5. (**E and F**) qRT-PCR quantification of PDCoV copy numbers in cytoplasmic lysates (Lys.) and supernatants (Sup.) of WT, A20 KO, and A20 OE cells. *n* = 3. (**G**) Representative immunofluorescence images showing colocalization of PDCoV with early endosomes (RAB5A^+^), late endosomes (RAB7B^+^), and lysosomes (LAMP2^+^) in WT, A20 KO, and A20 OE cells at 24 hpi. Nuclei were stained blue, PDCoV N protein was shown in green, and RAB5A, RAB7B, or LAMP2 were shown in yellow. Scale bar, 20 μm. Manders’ colocalization analysis of yellow and green fluorescence signals displayed as scatterplots. (**H**) Representative immunofluorescence images showing levels of viral dsRNA in WT, A20 KO, and A20 OE cells at 12 and 24 hpi. Scale bar, 20 μm. All experiments were representative of three or more independent experiments. Bars indicated mean + SD. **** *P* < 0.0001, *** *P* < 0.0005, ** *P* < 0.005, and * *P* < 0.05; ns, no significant difference.

Next, we asked about the impact of A20-mediated IPEC-J2 survival on PDCoV infection. At 24 hpi, A20 KO showed significantly lower levels of intracellular viral N protein compared to WT, while the N protein level in the supernatant was markedly higher (Fig. 4A). Overexpression of A20 (A20 OE) restored this distribution (Fig. 4A). Notably, the total level of N protein did not differ significantly across all treatment groups (Fig. 4A). For accurate quantification of viral distribution, RNA levels were further assessed. The results indicated that, although total viral output remained unchanged, A20 deficiency reduced intracellular viral accumulation and enhanced extracellular release (Fig. 4B), suggesting that the presence of A20 inhibited PDCoV release. To determine whether the released virus remained infectious, the viral titers in the supernatant were measured using the TCID_50_ assay. As expected, the infectious viral titer in the supernatant from A20 KO was significantly higher than that from WT (Fig. 4C), whereas A20 OE reduced viral release and resulted in lower titers (Fig. 4B and C). These results indicated that A20 did not influence PDCoV replication efficiency or infectivity, but instead altered the release pathway.

To elucidate the dynamics of PDCoV release, we first assessed membrane integrity in PDCoV-infected IPEC-J2 using propidium iodide (PI) staining. Significant PI uptake began at 18 hpi and peaked at 48 hpi (Fig. 4D), the timeline that closely aligned with the observed activation of PANoptosis (Fig. 1E). This suggested that viral release may depend on PANoptosis-mediated plasma membrane rupture. To test this hypothesis, we dynamically monitored the changes in intra- and extracellular viral loads. In WT, intracellular viral levels continuously accumulated (Fig. 4E), whereas extracellular viral levels showed an obvious increase after membrane rupture at 24 hpi (Fig. 4F), consistent with the timing of membrane rupture (Fig. 4D). These findings indicated a close association between viral release and membrane rupture. Notably, A20 deficiency accelerated the membrane rupture process, primarily occurring between 18 hpi and 48 hpi (Fig. 4D), resulting in a significant decrease in intracellular viral levels (Fig. 4E) and increased extracellular viral release at 24 and 48 hpi (Fig. 4F). In contrast, A20 overexpression reduced both cell death (Fig. 4D) and viral release (Fig. 4E and F), indicating that A20 restricted PDCoV release by regulating PANoptosis.

In order to exclude other potential contributions of A20, we examined the endosomal and lysosomal trafficking pathway prior to membrane rupture. At 12 hpi, PDCoV localization in early endosomes (RAB5A^+^), late endosomes (RAB7B^+^), or lysosomes (LAMP2^+^) was comparable among WT, A20 KO, and A20 OE (Fig. 4G), ruling out an effect of A20 on viral transport through the endosomal/lysosomal pathway. In addition, progeny virus production was assessed using double-stranded RNA staining. At 12 hpi, intracellular progeny virus was similar across WT, A20 KO, and A20 OE (Fig. 4H), indicating that A20 did not impair PDCoV replication. Unsurprisingly, by 24 hpi, A20 KO exhibited significantly reduced levels of intracellular progeny virus (Fig. 4H), while significant accumulation occurred in A20 OE, consistent with the observed viral release phenotypes (Fig. 4A and B). Together, these results demonstrated that A20 limited PDCoV release by suppressing PANoptosis, without affecting viral replication or endosomal trafficking.

### PDCoV release depended on plasma membrane rupture

**Fig 5:**
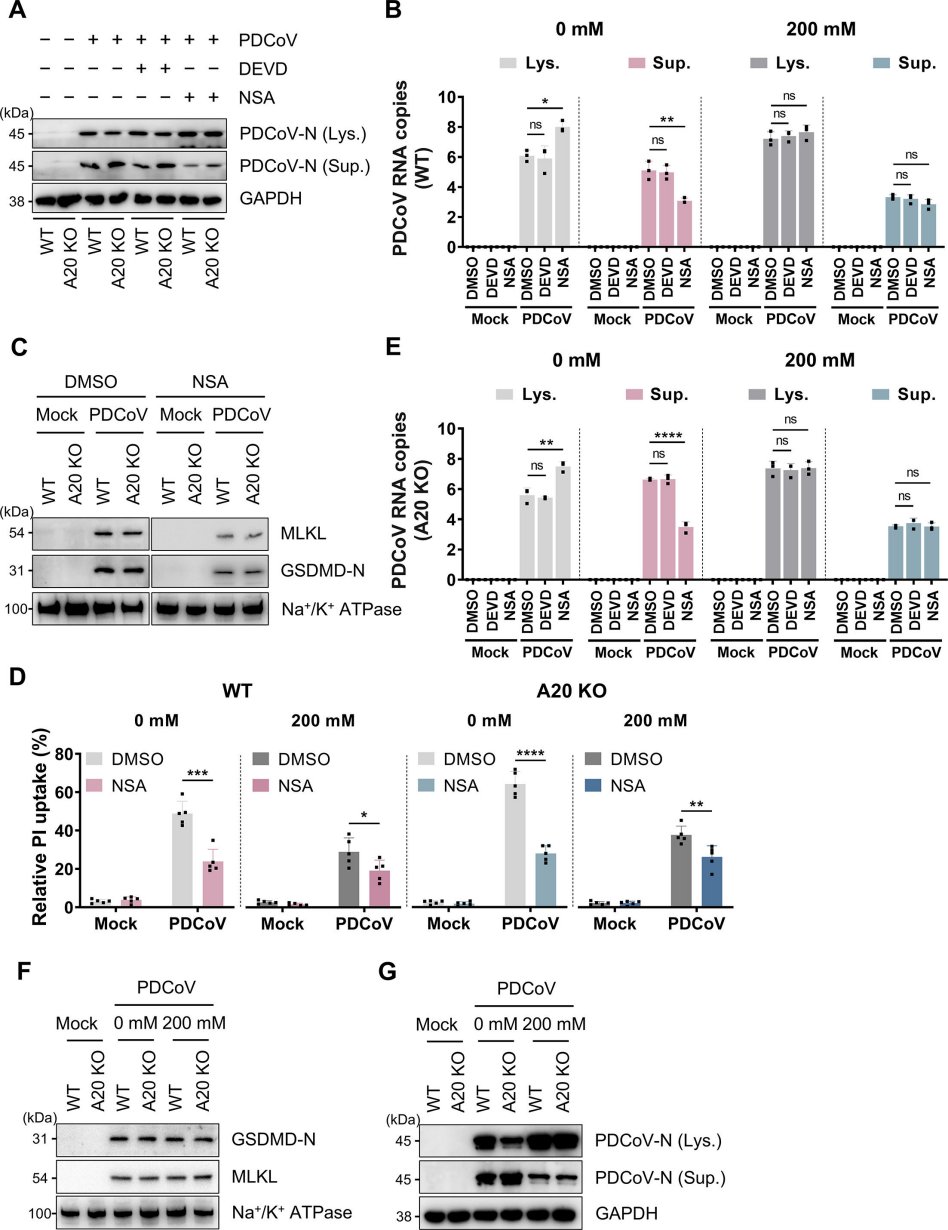
PDCoV release was dependent on lytic cell death. (**A**) Immunoblot analysis of PDCoV N protein levels in cytoplasmic lysates (Lys.) and supernatants (Sup.) of WT and A20 KO cells at 24 hpi (MOI of 0.5), with or without treatment with DEVD or NSA. Blots for GAPDH served as loading controls. (**B**) qRT-PCR quantification of PDCoV N gene copy numbers in cytoplasmic lysates (Lys.) and supernatants (Sup.) of WT cells with or without 200 mM mannitol treatment. *n* = 3. (**C**) Immunoblot analysis of membrane-associated MLKL and GSDMD-N in WT and A20 KO cells at 24 hpi, with or without NSA treatment. Na^+^/K^+^ ATPase was used as a membrane protein loading control. (**D**) Kinetics of PI-permeable cell death in WT and A20 KO cells at 24 hpi with or without 200 mM mannitol treatment. *n* = 6. (**E**) qRT-PCR quantification of PDCoV N gene copy numbers in cytoplasmic lysates (Lys.) and supernatants (Sup.) of A20 KO cells, with or without 200 mM mannitol treatment. *n* = 3. (**F**) Immunoblot analysis of membrane-associated MLKL and GSDMD-N in WT and A20 KO cells at 24 hpi, with or without 200 mM mannitol treatment. Na^+^/K^+^ ATPase was used as a loading control. (**G**) Immunoblot analysis of PDCoV N protein levels in cytoplasmic lysates (Lys.) and supernatants (Sup.) of WT and A20 KO cells at 24 hpi, with or without 200 mM mannitol treatment. Blots for GAPDH served as loading controls. All experiments were representative of three or more independent experiments. Bars indicated mean + SD. **** *P* < 0.0001, *** *P* < 0.0005, ** *P* < 0.005, and * *P* < 0.05; ns, no significant difference.

For determining the contribution of different PCD pathways within PANoptosis to PDCoV release, we used the specific caspase-3 inhibitor Z-DEVD-FMK (DEVD) to block apoptosis in both WT and A20 KO. The results showed that the inhibitor treatment did not significantly affect viral release into the supernatant compared to the control (Fig. 5A and B). This suggested that apoptosis was not a major factor in PANoptosis-mediated viral release. Mechanistically, GSDMD-N and pMLKL mediate the formation of transmembrane pores, which disrupt cellular osmotic balance and ultimately lead to membrane rupture. To interfere with their function, we applied necrosulfonamide (NSA), a dual-target inhibitor of GSDMD-N and pMLKL. Membrane protein fractionation experiments confirmed that NSA treatment effectively inhibited the membrane-associated forms of GSDMD-N and pMLKL (Fig. 5C). Correspondingly, PI uptake was significantly reduced (Fig. 5D), validating the inhibitory effect of NSA on pore formation. Functionally, NSA treatment significantly suppressed PDCoV release in WT and was accompanied by intracellular accumulation of viral N protein (Fig. 5A and B). In A20 KO, NSA also successfully reversed the enhanced viral release phenotype caused by gene deletion (Fig. 5A and E). These findings suggested that GSDMD-N and pMLKL-mediated pore formation was a core mechanism driving PDCoV release.

To distinguish the relative contributions of pore-dependent and membrane rupture-dependent release, IPEC-J2 was infected with PDCoV under hyperosmotic conditions supplemented with 200 mM mannitol. This treatment provided an additional 200 mOsm of osmolarity to counteract membrane rupture (Fig. 5D), without affecting the membrane localization of GSDMD-N and pMLKL (Fig. 5F). Under this model, the level of N protein in the supernatant was significantly reduced in WT (Fig. 5G). Consistent with viral RNA quantification results, mannitol inhibited the release of a majority of viral particles (Fig. 5B). This suppressive effect was also observed in A20 KO (Fig. 5G and E). Notably, the addition of NSA to WT (Fig. 5B) and A20 KO (Fig. 5E) under hyperosmotic conditions produced only limited further inhibition, indicating that pore-mediated viral release alone is inefficient. Collectively, these results demonstrated that PDCoV release primarily depended on late-stage PANoptosis-mediated plasma membrane rupture rather than direct transport through membrane pores.

### Inhibition of PANoptosis reduced the spread of PDCoV progeny virus

**Fig 6:**
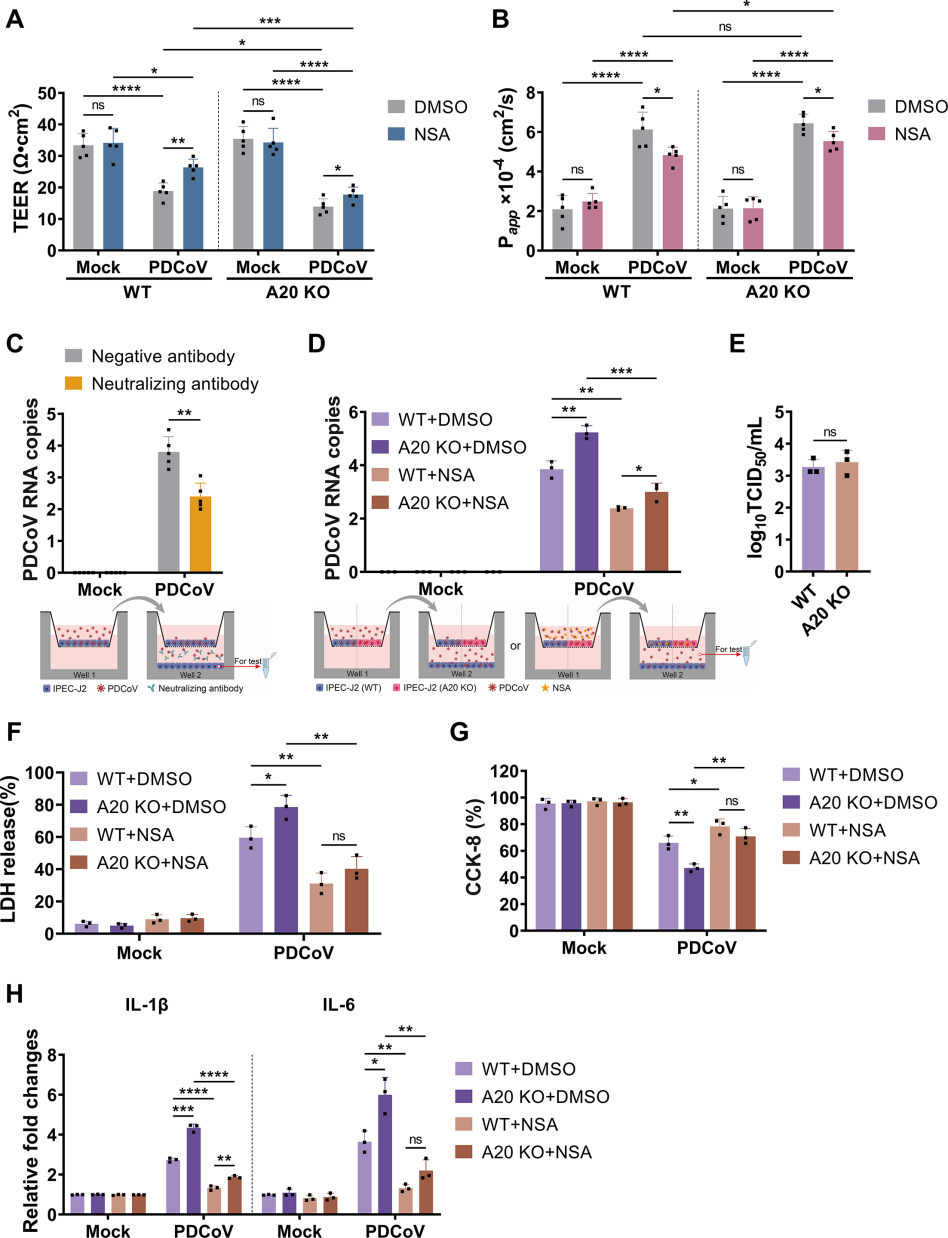
NSA inhibited the spread of PDCoV. The *in vitro* intestinal barrier model was established using IPEC-J2 cells. At 24 hpi, the model was evaluated for transepithelial electrical resistance (TEER) (**A**, *n* = 6) and NaFI permeability (**B**, *n* = 6). (**C**) qRT-PCR analysis of PDCoV N gene copy numbers in the basal compartment cells in the presence or absence of neutralizing antibodies. *n* = 5. (**D**) qRT-PCR analysis of PDCoV N gene copy numbers in the lower chamber with or without NSA treatment. *n* = 3. (**E**) TCID_50_ assay of PDCoV titers in the lower chamber of WT and A20 KO models. *n* = 3. (**F and G**) Cytotoxicity (F, *n* = 3) and cell viability (G, *n* = 3) assays of basal compartment cells in WT and A20 KO models with or without NSA treatment. (**H**) mRNA expression levels of IL-1β and IL-6 in basal compartment cells of WT and A20 KO models with or without NSA treatment. *n* = 3. All experiments were representative of three or more independent experiments. Bars indicated mean + SD. **** *P* < 0.0001, *** *P* < 0.0005, ** *P* < 0.005, and * *P* < 0.05; ns, no significant difference.

E-cadherin was markedly downregulated in intestinal epithelial cells following PDCoV infection, indicative of intestinal barrier disruption (Fig. 1J). To investigate the impact of PANoptosis on intestinal barrier function, we established a monolayer intestinal epithelial model using IPEC-J2 cultured on Transwell inserts. Barrier integrity was assessed by transepithelial electrical resistance (TEER), and permeability was evaluated using the fluorescent tracer sodium fluorescein (NaFI). PDCoV infection led to a significant decline in barrier integrity over time (Fig. 6A), and this effect was further exacerbated by A20 deficiency (Fig. 6A). This indicated that PANoptosis contributed to intestinal barrier damage. As a result, barrier permeability significantly increased (Fig. 6B). Treatment with NSA partially restored barrier function (Fig. 6A and B), suggesting that A20 could maintain intestinal barrier homeostasis by inhibiting PANoptosis.

For exploring the role of PANoptosis in viral dissemination, a co-culture model was established by seeding wild-type IPEC-J2 in the bottom chamber of the Transwell system. In this model, the addition of neutralizing antibodies to both WT and A20 KO nearly abolished infection of the bottom-layer cells (Fig. 6C), indicating that PDCoV spread across cells depends on the release of free viral particles. In the A20 KO model, the viral load in the lower chamber was significantly higher than in the WT model (Fig. 6D). This indicated that A20 deficiency promoted viral release and applied to expanding the spread of progeny viruses. Viruses collected from the lower chamber of both WT and A20 KO models were used to infect cells at the same multiplicity of infection (MOI). The TCID_50_ assays revealed no significant differences in infectivity between the two groups (Fig. 6E), confirming that the increased spread resulted from increased release rather than enhanced infectivity. In contrast, NSA treatment reduced viral release (Fig. 6D) in both WT and A20 KO models, further reinforcing the critical role of PANoptosis in promoting viral dissemination.

Basolateral cells that were infected by virus derived from A20 KO cultures exhibited higher cytotoxicity (Fig. 6F) and lower viability (Fig. 6G), both of which were mitigated by NSA treatment (Fig. 6F and G). However, it is important to note that NSA reduced the expression of inflammatory cytokines in these cells (Fig. 6H), likely due to its targeted inhibition of pyroptosis and necroptosis. Collectively, these findings identified A20 as a key host factor that regulated PANoptosis to restrict the spread of PDCoV. Therapeutic strategies that target lytic cell death may offer promising antiviral potential by restricting viral dissemination.

## DISCUSSION

**Fig 7:**
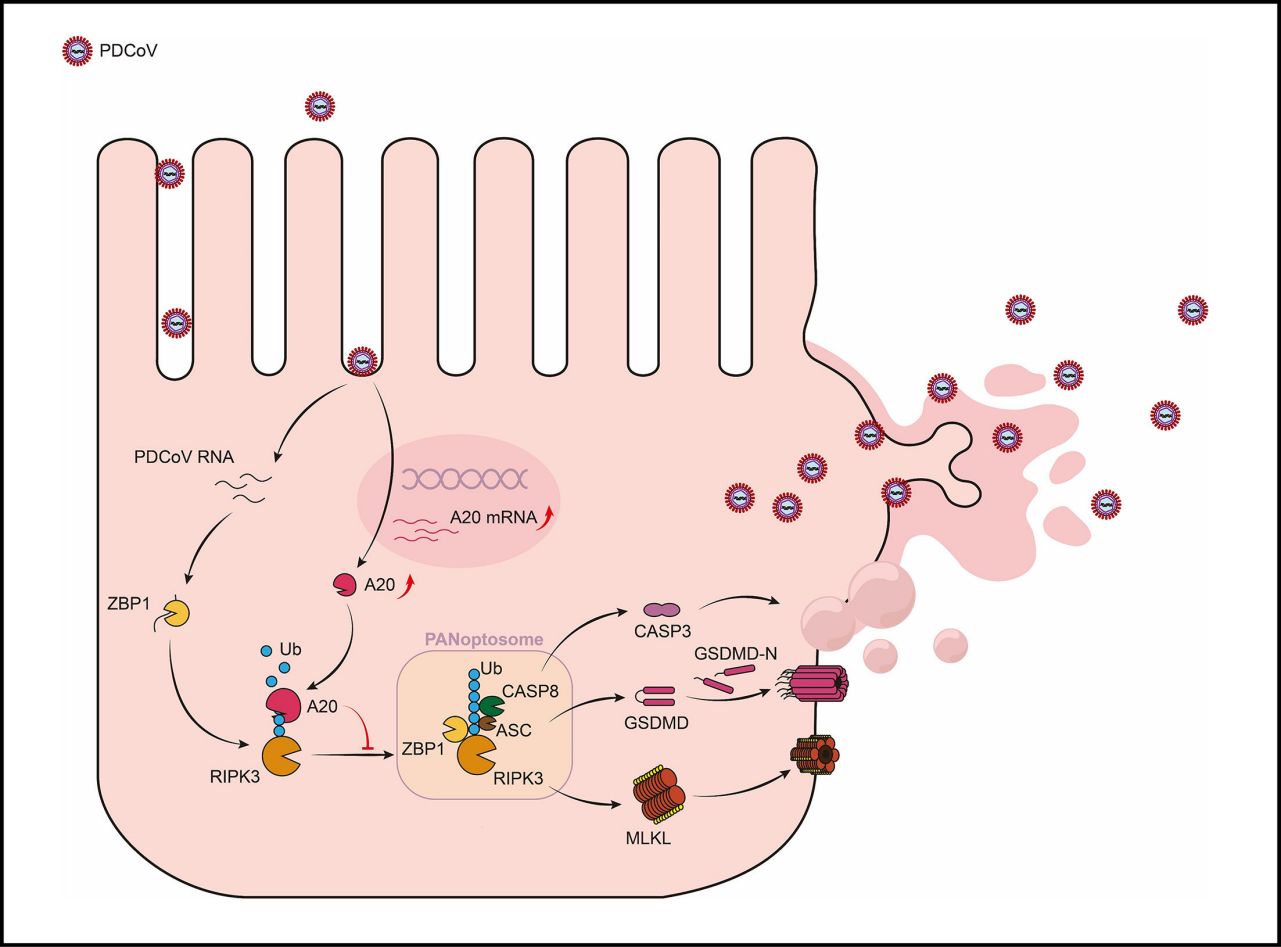
A20 restricted PDCoV release by suppressing PANoptosis-mediated plasma membrane rupture. Upon infecting intestinal epithelial cells, PDCoV RNA is sensed by ZBP1, triggering RIPK3 ubiquitination and PANoptosome assembly. This initiates PANoptosis, where GSDMD and MLKL pores cause osmotic imbalance and plasma membrane rupture, facilitating viral release. The host protein A20 counteracts this by deubiquitinating RIPK3 to suppress the PANoptosome, thereby delaying lysis and restricting viral spread.

The role of PCD in antiviral immunity has attracted increasing attention. This study systematically elucidated the molecular mechanism by which PDCoV infection induced PANoptosis in porcine intestinal epithelial cells and demonstrated that this process facilitated viral release through plasma membrane rupture, thereby significantly compromising the integrity of the intestinal barrier (Fig. 7). This finding echoes the study by Karki et al., which reported PANoptosis in alveolar type II epithelial cells induced by SARS-CoV-2 (36). These results suggest that different coronaviruses may drive tissue damage through the coordinated activation of multiple cell death pathways (37). This common mechanism may represent a key strategy by which coronavirus infections initiate inflammatory pathological responses.

As a classical negative regulator of inflammation, A20 displays diverse functions across different viral infections. In infections caused by HCV and IAV, A20 upregulation has been shown to promote viral replication (38, 39). However, our data showed that although A20 expression was significantly elevated during PDCoV infection and increased intracellular viral load, it did not affect PDCoV replication efficiency. Instead, it modulated the host antiviral response. The conclusion that the regulation of cell death influences viral load was also supported by the findings of Liuyang Du et al. (40). We further demonstrated that this phenomenon resulted from A20-mediated feedback inhibition of the NF-κB pathway, along with its regulation of PANoptosome assembly and activation via modulation of RIPK3 ubiquitination. This discovery broadens our understanding of the role of A20 in viral infection and suggests that it may exert pathogen-specific regulatory functions in antiviral immunity. The host upregulates A20 to establish a defensive barrier that restricts the disease process by preventing excessive cell death. This mechanism has attracted attention in various disease models. Previous studies have shown that A20 can suppress the RIPK3-MLKL pathway to maintain cellular homeostasis (35, 41, 42). Taken together, these findings indicate that A20 not only plays a critical role in balancing inflammatory responses but also influences cell fate through the A20-RIPK3 ubiquitination axis.

Apoptosis, pyroptosis, and necroptosis play complex roles in antiviral defense and inflammation regulation. Recent studies have shown that PDCoV induces both apoptosis and pyroptosis (43–46). Previous studies have shown that PDCoV-induced pyroptosis was more pronounced in the duodenum than in other intestinal segments (46). Specifically, PDCoV could simultaneously activate pyroptosis mediated by both GSDMD and GSDME. The viral nsp5 protein selectively cleaved GSDMD to eliminate its antiviral effect while preserving GSDME-dependent pyroptotic activity (47). This differential regulation led to significantly lower viral loads in the duodenum compared to the jejunum and ileum (48–50), highlighting the critical role of lytic cell death in clearing infected cells. However, our data indicated that during the later stages of infection, PDCoV might exploit this form of cell death to facilitate its release. Although lytic death pathways such as pyroptosis contributed to viral clearance (47), the associated plasma membrane rupture not only promoted the dissemination of viral particles but might also aggravate local inflammation due to the release of intracellular contents. Notably, this strategy was similar to that of norovirus, which encoded an MLKL-like protein to induce necroptosis and facilitate viral release (51). These suggested a shared evolutionary mechanism through which viruses utilize lytic cell death to enhance their spread. Furthermore, although PDCoV had been shown to induce apoptosis and thereby facilitate viral release (52), our findings demonstrated that PANoptosis induced more extensive membrane rupture and increased viral egress more effectively than apoptosis alone, thereby offering a greater advantage for viral dissemination. This discovery challenges the view that coronaviruses are released through exocytosis and cell-cell fusion (53, 54). Based on these findings, we hypothesize that PDCoV may rely on exocytosis during the early phase of infection but gradually shift toward membrane rupture-driven release as PANoptosis becomes activated in later stages. This dynamic transition may represent an adaptive strategy by which PDCoV optimizes its replication and transmission within the intestinal environment. This model is further supported by ultrastructural analyses of PDCoV (54). Collectively, these findings suggested that targeting cell death-associated release pathways could offer new therapeutic strategies to limit PDCoV spread.

Our study highlighted that PANoptosis-dependent release of progeny PDCoV virions primarily depended on the extent of plasma membrane rupture caused by osmotic stress, rather than on mechanical passage through pores formed by pore-forming proteins. Pores formed by GSDMD-N oligomerization measure approximately 10–20 nm (55, 56), allowing the passage of dextran molecules ranging from 3 to 20 kDa or larger (55), yet still significantly smaller than PDCoV virions, which are about 58–70 nm in diameter (54). Although structural data on MLKL-mediated pores are limited, existing evidence indicates that these pores are even smaller, permitting only dextrans smaller than 10 kDa to pass through (57). Therefore, the size of PDCoV virions far exceeds the permeability limits of pores formed by GSDMD-N or pMLKL, suggesting that viral particles are unlikely to be released through these channels. These findings further support the conclusion that PDCoV progeny virions are not released through channels formed by pore-forming proteins. Based on this mechanism, we found that inhibition of PANoptosis, targeting pore-forming proteins such as GSDMD or MLKL, or application of osmoprotective strategies each significantly reduce viral release. It is noteworthy that hyperosmotic environments regulate membrane rupture via different mechanisms. Mannitol-induced hyperosmotic stress alleviates membrane rupture by restoring osmotic pressure without affecting GSDMD activation (58). In contrast, hyperosmotic stress induced by NaCl or KCl not only modulates osmotic pressure but also inhibits GSDMD cleavage and pore formation (58). To clearly distinguish pore-dependent from rupture-dependent viral release, we selected mannitol as an experimental control. However, for potential therapeutic applications, NaCl or KCl may provide more potent inhibition of viral release.

In summary, this study elucidated the molecular mechanism by which PDCoV promoted viral release by inducing PANoptosis in intestinal epithelial cells. The host protein A20 restricted excessive cell death by suppressing PANoptosome assembly via modulation of RIPK3 ubiquitination. PDCoV release primarily depended on late-stage PANoptosis-mediated plasma membrane rupture. Inhibition of this process significantly reduced viral transmission and improved intestinal barrier integrity. These findings uncovered a novel strategy by which PDCoV exploited host cell death pathways to facilitate its spread and provide a theoretical basis for developing targeted antiviral interventions.

## MATERIALS AND METHODS

### Animal infections

Healthy piglets were randomly divided into control and infected groups. Piglets were orally inoculated with PDCoV at 2 × 10^5^ TCID_50_/mL or an equal volume of Dulbecco’s modified Eagle’s medium (DMEM) per piglet. At 48 hpi, jejunal tissues were processed for immunofluorescence assay and molecular detection.

### Cells and viruses

The IPEC-J2, LLC-PK1, and HEK293T cells used in this study were purchased from the China Center for Type Culture Collection (CCTCC). A20 KO and RIPK3 K5A cells were generated using the CRISPR/Cas9 gene editing system. The cell lines were cultured in DMEM (Gibco, USA) supplemented with 10% fetal bovine serum (Gibco, USA) and 1% (vol/vol) penicillin/streptomycin (Solarbio, China) at 37°C with 5% CO_2_. The PDCoV CZ2020 strain (GenBank accession number: OK546242) was isolated from a piglet suffering from severe diarrhea and passaged in LLC-PK1 cells with DMEM supplemented with 7.5 µg/mL trypsin.

### Generation of gene-edited IPEC-J2 by CRISPR/Cas9

The single guide RNA (sgRNA) targeting A20 (GenBank ID: NM_001267890.1) or non-targeting sgRNA were cloned into lentiCRISPRv2 puro vector (Addgene, USA) following the protocol from the Zhang Feng laboratory (59), with an additional G added at the beginning of the sgRNA sequence. The lentiviral packaging system consisted of LentiCRISPRv2 puro, pCMV-VSV-G (Addgene, USA), and psPAX2 (Addgene, USA), which were transfected into HEK293T cells using Lipofectamine 3000 transfection reagent (Thermo Fisher Scientific, USA). Viral supernatant was collected 60 h post-transfection, filtered, and concentrated to obtain lentiviral particles, followed by determination of viral titer. IPEC-J2 cells were infected with the lentivirus at an MOI of 10 in the presence of 5 μg/mL polybrene (MedChemExpress, USA). Stable stress-resistant cells were selected using 1 μg/mL puromycin (MedChemExpress, USA), and monoclonal cell lines were established by limiting dilution. Among multiple sgRNA candidates, the most efficient sequence (sgRNA: GTATTTGAGCAACATGCGGA) was identified by western blot analysis and used for subsequent experiments.

For the construction of the RIPK3 mutant, the mRNA sequence containing the position 5 mutation was cloned into the pcSLenti-EF1-EGFP-F2A-BSR-CMV-MCS-3×FLAG-WPRE vector. For the construction of the A20 OE, the mRNA sequence was cloned into the pcSLenti-EF1-EGFP-F2A-BSR-CMV-MCS-3×FLAG-WPRE vector. Lentivirus was generated as previously described and used to infect RIPK3-knockout or WT IPEC-J2 cells at an MOI of 10. Stable stress-resistant cell populations were selected using 10 μg/mL blasticidin S (MedChemExpress, USA). FLAG-tagged expression was confirmed by WB.

### Cell stimulations

IPEC-J2 WT, A20 KO, A20 OE, or RIPK3 K5A cells were seeded into culture plates and incubated until a confluent monolayer was formed. Cells were counted before all infection assays to ensure an accurate MOI. PDCoV was inoculated at an MOI of 0.5 in the presence of 7.5 μg/mL trypsin, allowing viral adsorption for 1 h. Samples were collected at designated time points for further analysis. For inhibitor-related experiments, cells were pretreated with 20 μM Z-DEVD-FMK (MedChemExpress, USA) or 5 μM necrosulfonamide (MedChemExpress, USA) at 37°C with 5% CO_2_ for 0.5 h, followed by co-incubation with PDCoV until the indicated time points. The preparation of inactivated PDCoV was performed as described (60). For osmotic protection assays, cells were cultured and infected in DMEM supplemented with 200 mM mannitol. Supernatants were collected for lactate dehydrogenase (LDH) release assays. Cell lysates were subjected to western blot analysis for the given protein detection. RNA was extracted to analyze the transcriptional levels of target genes.

### Cell viability and cytotoxicity assays

IPEC-J2 WT, A20 KO, or A20 OE cells were seeded into 96-well plates and cultured until a confluent monolayer was formed. PDCoV was inoculated at an MOI of 0.5 in the presence of 7.5 μg/mL trypsin, allowing viral adsorption for 1 h. Infections were continued until the indicated time points. LDH release and cell viability were assessed using an LDH Cytotoxicity Colorimetric Assay Kit (Beyotime Biotechnology, China) and a CCK-8 assay kit (Beyotime Biotechnology, China), respectively, following the manufacturer’s instructions. LDH release was expressed as a percentage of total LDH (100% lysis), and cell viability was presented as a percentage relative to uninfected cells. Culture medium without cells was used to subtract background absorbance.

### Immunoblotting and co-immunoprecipitation

IPEC-J2 WT, A20 KO, A20 OE, or RIPK3 K5A cells were seeded into six-well plates and cultured until a confluent monolayer was formed. PDCoV was inoculated at an MOI of 0.5 in the presence of 7.5 μg/mL trypsin, allowing viral adsorption for 1 h. Infections were continued until the indicated time points. Cells were lysed in RIPA buffer containing 1 mM phenylmethylsulfonyl fluoride. For the detection of phosphorylated proteins, phosphatase inhibitor cocktail 2 was additionally added. Lysates were mixed with SDS-PAGE polyclonal loading buffer and incubated at 95°C  for 10 min, followed by centrifugation at 12,000   *g* for 5 min at 4 °C. A total of 15 μg of supernatant protein was loaded onto a 12% SDS-PAGE. Proteins were transferred onto 0.22 μm or 0.45 μm PVDF membranes, depending on the molecular weight of the target protein. After blocking, membranes were incubated overnight at 4°C  with anti-PDCoV-mAb (prepared by our lab, 1:2,000), rabbit anti-GSDMD-NT polyclonal antibody (Bioworld, BS67358, 1:2,000), anti-caspase-3 pAb (Evonab, bs-0081R, 1:2,000), anti-MLKL mAb (ABclonal, A10507, 1:2,000), phospho-MLKL-S358 rabbit pAb (ABclonal, AP1244, 1:500), NF-κB p65/RelA rabbit mAb (ABclonal, A19653, 1:10,000), phospho-NF-κB p65/RelA-S536 rabbit mAb (ABclonal, AP1294, 1:10,000), TNFAIP3 monoclonal antibody (Proteintech, 66695-1-Ig, 1:4,000), ubiquitin polyclonal antibody (Proteintech, 10201-2-AP, 1:8,000), rabbit anti-GAPDH polyclonal antibody (Bioworld, AP0063, 1:10,000), Na^+^/K^+^-ATPase rabbit mAb (ABclonal, A11683, 1:100,000), RIPK3 polyclonal antibody (Proteintech, 29080-1-AP, 1:5,000), caspase-8/P43/P18 polyclonal antibody (Proteintech, 13423-1-AP, 1:1,000), ASC/TMS1 polyclonal antibody (Proteintech, 10500-1-AP, 1:5,000), and ZBP1 polyclonal antibody (Proteintech, 13285-1-AP, 1:4,000). PVDF membranes were incubated with goat anti-rabbit (Bioworld, BS13278, 1:50,000) or goat anti-mouse (Bioworld, BS12478, 1:50,000) at room temperature for 1 h. Protein bands were visualized using enhanced chemiluminescence reagents.

For co-immunoprecipitation assays, cells were lysed in NP-40 buffer supplemented with protease inhibitors. Lysates were centrifuged at 12,000   *g* for 5 min at 4°C. The resulting supernatant was incubated with protein A/G agarose beads pre-bound to 2 μg of the indicated antibody at room temperature for 30 min. Beads were washed five times with NP-40 buffer. The precipitates were boiled in SDS-PAGE loading buffer at 95°C for 10 min and subjected to western blotting as described above. Secondary antibodies used for Co-IP detection were horseradish peroxidase (HRP)-conjugated goat anti-mouse IgG heavy chain (ABclonal, AS064, 1:5,000), HRP-conjugated goat anti-rabbit IgG heavy chain (Abbkine, A25012, 1:5,000), and HRP-conjugated goat anti-mouse IgG light chain (ABclonal, AS062, 1:5,000).

### Immunofluorescence assay

IPEC-J2 WT, A20 KO, or A20 OE cells were seeded in confocal dishes and infected with PDCoV for 24 h, unless otherwise specified. Cells were fixed with 5% paraformaldehyde at room temperature for 30 min and washed three times with PBS. Permeabilization was performed with 0.1% Triton X-100 for 5 min, followed by blocking with 5% BSA at room temperature for 1 h. Cells were incubated overnight at 4°C with primary antibodies against the target proteins. After three PBS washes, cells were incubated for 1 h at room temperature in the dark with Goat Anti-Rabbit IgG H&L (Alexa Fluor 647; Abcam, ab150083, 1:1,000) and Goat Anti-Mouse IgG H&L (Alexa Fluor 488; Abcam, ab150113, 1:1,000). Nuclei were counterstained with DAPI. Images were acquired using a confocal laser scanning microscope (ZEISS, LSM 800) and analyzed for colocalization using ImageJ software.

### Quantitative real-time PCR

Total RNA was isolated from cells or tissues using the FastPure Cell/Tissue Total RNA Isolation Kit (Vazyme, RC101-01) according to the manufacturer’s instructions. Residual genomic DNA was removed using HiScript II Reverse Transcriptase (Vazyme, R233-01), and cDNA was synthesized by reverse transcription reaction. Quantitative real-time PCR (qRT-PCR) was performed using SYBR GREEN PCR in StepOnePlus Real-Time PCR system (ThermoFisher Scientific, Applied Biosystems) using 40 cycles of 95°C for 5 min, 95°C for 10 s, and 60°C for 30 s. ∆∆CT method and housekeeping gene GAPDH expression were used to determine the expression level of target genes. Absolute quantitative PCR was employed for PDCoV detection. Primers for RT-PCR were listed in Table 1.

**TABLE 1:** Oligonucleotides

Gene	Sequence (5′−3′)
IL-1β-F	AACGTGCAGTCTATGGAGT
IL-1β-R	GAACACCACTTCTCTCTTCA
IL-6-F	CTGGCAGAAAACAACCTGAACC
IL-6-R	TGATTCTCATCAAGCAGGTCTCC
A20-F	AATGGGACAACCTTATCAA
A20-R	GAACCCGATTCCAAACT
PDCoV-N-F	ATCGACCACATGGCTCCAA
PDCoV-N-R	CAGCTCTTGCCCATGTAGCTT
GAPDH-F	CCTTCCGTGTCCCTACTGCCAAC
GAPDH-R	GACGCCTGCTTCACCACCTTCT

### PI analysis of cell membrane integrity

IPEC-J2 WT, A20 KO, and A20 OE cells were seeded into 96-well plates on the bottom of black transparent glass and cultured until a confluent monolayer was formed. PDCoV was inoculated at an MOI of 0.5 in the presence of 7.5 μg/mL trypsin, allowing viral adsorption for 1 h. Cells treated with 0.5% Triton X-100 served as the positive control. Wells containing medium only were used as blank controls for background subtraction. Cells were incubated in DMEM containing PI at a final concentration of 10 μg/mL until the indicated time points. Fluorescence intensity was measured using a multimode microplate reader (TECAN, Spark) with excitation at 535 nm and emission at 615 nm. Results were expressed as a percentage of total PI uptake.

### TCID_50_ assays

LLC-PK1 was seeded into 96-well plates and cultured until a confluent monolayer was formed. Viral samples were serially diluted in DMEM supplemented with 7.5 μg/mL trypsin, and each dilution was applied to eight replicate wells. Plates were incubated at 37°C with 5% CO_2_ for 96 h. Wells exhibiting cytopathic effects were recorded as positive for viral infection. Viral titers were determined using the Reed-Muench method.

### Transwell model and indirect co-culture assay

Cell culture inserts (Corning, CLS3470) were pre-coated with collagen (Sigma, 11179179001) for 4 h, then placed into 24-well plates containing 500 μL of culture medium in the lower chamber. IPEC-J2 WT or A20 KO cells were seeded into the apical chamber and cultured until a confluent monolayer was formed. For TEER measurement, cells were infected with PDCoV at an MOI of 0.5 in the presence of 7.5 μg/mL trypsin for 1 h, followed by continued incubation for 24 h. TEER was measured using an EVOM2 epithelial voltohmmeter equipped with STX2 electrode to assess resistance across the apical and basolateral chambers. Inserts without cells were used as blank controls, and their resistance values were subtracted from experimental readings. The TEER value (Ω·cm^2^) was obtained by multiplying the measured resistance (Ω) by the membrane surface area (cm^2^). For the NaFI permeability assay, 1% NaFI was added to the apical chamber and incubated for 10 min at 37°C with 5% CO_2_ following PDCoV infection for the indicated time. Samples were then collected from the basolateral chamber, and fluorescence was measured using excitation at 460 nm and emission at 520 nm. The permeability coefficient (*P_app_*) was determined by measuring the increase in fluorescence intensity in the basolateral chamber over a defined time period. The rate of fluorescence change was normalized to the volume of the basolateral compartment, the surface area of the cell monolayer, and the initial fluorescence intensity in the apical chamber. *P_app_* reflects the rate of NaFI diffusion per unit membrane area and per unit initial concentration.

For the indirect co-culture assay, IPEC-J2 WT cells were first seeded into 24-well plates and cultured until a confluent monolayer was formed. Viral infection was restricted to the upper-chamber cells. Infected IPEC-J2 WT or A20 KO cell inserts were then transferred into the wells for non-contact co-culture. At 24 hpi, culture supernatants or basolateral cells were collected for subsequent analyses. Regarding the use of neutralizing antibodies, they were added to the basal-chamber medium simultaneously with the transfer of the insert. For pharmacological intervention experiments, NSA was added only to the medium in the upper chamber and applied simultaneously with viral infection. At the end of the designated treatment period, the insert was transferred to fresh medium without NSA and placed over pre-seeded bottom-layer cells. Co-culture was then continued for 24 h, after which the bottom-layer cells were collected for analysis.

### Statistical analyses

The data in this study were representative of at least three independent experiments. All statistical analyses were performed using GraphPad Prism for two-tailed unpaired Student’s *t*-tests or one-way analysis of variance. All data were represented as mean ± standard deviation. Differences were considered statistically significant when *P* < 0.05, where * *P* < 0.05, ** *P* < 0.005, *** *P* < 0.0005, and **** *P* < 0.0001; ns, no significant difference.

## Data Availability

The relevant data supporting the results of this study can be found in the article.
